# Using Large Language Models to Enhance the Reusability of Sensor Data

**DOI:** 10.3390/s24020347

**Published:** 2024-01-06

**Authors:** Alberto Berenguer, Adriana Morejón, David Tomás, Jose-Norberto Mazón

**Affiliations:** Department of Software and Computing Systems, University of Alicante, Carretera San Vicente del Raspeig s/n, 03690 San Vicente del Raspeig, Spain; aberenguer@dlsi.ua.es (A.B.); adriana.morejon@ua.es (A.M.); dtomas@dlsi.ua.es (D.T.)

**Keywords:** Internet of Things, sensor data, interoperability, data reusability, data processing

## Abstract

The Internet of Things generates vast data volumes via diverse sensors, yet its potential remains unexploited for innovative data-driven products and services. Limitations arise from sensor-dependent data handling by manufacturers and user companies, hindering third-party access and comprehension. Initiatives like the European Data Act aim to enable high-quality access to sensor-generated data by regulating accuracy, completeness, and relevance while respecting intellectual property rights. Despite data availability, interoperability challenges impede sensor data reusability. For instance, sensor data shared in HTML formats requires an intricate, time-consuming processing to attain reusable formats like JSON or XML. This study introduces a methodology aimed at converting raw sensor data extracted from web portals into structured formats, thereby enhancing data reusability. The approach utilises large language models to derive structured formats from sensor data initially presented in non-interoperable formats. The effectiveness of these language models was assessed through quantitative and qualitative evaluations in a use case involving meteorological data. In the proposed experiments, GPT-4, the best performing LLM tested, demonstrated the feasibility of this methodology, achieving a precision of 93.51% and a recall of 85.33% in converting HTML to JSON/XML, thus confirming its potential in obtaining reusable sensor data.

## 1. Introduction

Internet of Things (IoT) infrastructures generate an abundance of data through devices equipped with diverse sensors. However, the unexploited potential of these data to drive innovative data-driven products and services persists due to the dependence on sensor manufacturers and individual companies for data description, storage, and transmission, hindering seamless sharing and comprehension by third parties [1].

Efforts to address this issue, such as the European Data Act (https://eur-lex.europa.eu/legal-content/EN/TXT/?uri=COM:2022:68:FIN, accessed on 20 September 2023), aim to regulate data accessibility advocating for high-quality access to sensor-generated data. The proposed European regulation emphasises that data owners must ensure the accuracy, completeness, reliability, relevance, and currency of data shared with third parties, while also respecting trade secrets or intellectual property rights.

However, despite initiatives enabling access to sensor-generated data, interoperability challenges impede the reusability of such data [2]. Sensor data often gets shared in HTML format, necessitating laborious, resource-intensive, and error-prone data processing for conversion into reusable formats like JSON or XML. Notably, there are challenges for making sensor data more interoperable [2]:

- Sensor data exhibit heterogeneous characteristics, impeding the establishment of standardised data models essential for improving interoperability. Consequently, sensor data often reside in arbitrary, frequently proprietary formats.
- Anticipated applications of sensor data remain unknown a priori, necessitating data formats that facilitate unrestricted and interoperable reuse, rather than catering solely to specific applications.
- Variations in architecture and network configurations across distinct sensor systems pose a hurdle. Consequently, manual adaptations are requisite for sensor data acquisition in each scenario to improve interoperability.

As a result, sensor data remains inaccessible and unprocessable by machines, making it difficult to reuse [3], as it is often shared in non-interoperable formats like HTML [4] in web portals.

This study introduces a methodology designed to convert raw sensor data obtained from web portals into structured, more easily interpretable, and reusable formats (e.g., JSON, XML, or CSV). The specific objectives pursued in this study are:

1. Enable users to interact by stating their objectives, thereby obtaining the necessary sensor data to meet these objectives.
2. Provide immediate access to sensor data published in unstructured formats by converting it into interoperable formats, facilitating easier reuse of the data.
3. Achieve a high level of automation regardless of the diversity of topics that sensor data can cover.

The methodology is driven by the necessity to render sensor data interoperable, thereby maximising its reuse and addressing the challenges mentioned previously. This approach prioritises the inclusion of reusers and their information needs as a central component of the methodology. In practice, reusers need only to specify their objectives in relation to sensor data applications. Subsequently, the retrieval of the structured sensor data required to fulfil these objectives is automated using LLMs on a selection of web portals. Specifically, the methodology comprises four key steps: (i) stating specific objective requiring sensor data, (ii) identifying the portals hosting the data, (iii) devising data retrieval procedures, and (iv) leveraging large language models (LLMs) [5] to generate structured standardised data formats (such as JSON or XML) from raw sensor data initially in non-interoperable formats (like HTML). This methodology is designed to facilitate the consumption of sensor data initially published in unstructured formats, such as HTML. Consequently, the scope of this proposal is specifically limited to certain web portals that support sensor discovery in IoT networks. An example is Censys (see Section 2.2), known for its extensive publication of sensor data in HTML format.

The methodology was applied and tested through a specific use case utilising meteorological data sourced from a web portal hosting sensors. The evaluation conducted gauged the precision and recall of various state-of-the-art LLMs in converting HTML sensor data into XML format.

The remainder of this paper is organised as follows: Section 2 presents an overview of related work encompassing LLMs and IoT platforms; Section 3 details the proposed methodology for extracting structured information from raw sensor data; Section 4 outlines a specific use case revolving around meteorological data; Section 5 delves into evaluation, testing the performance of different LLMs to convert sensor data in HTML into XML format; and finally, Section 7 provides concluding remarks and outlines potential future research directions.

## 2. Related Work

This section describes the related work within the domain of LLMs and offers insights into existing web portals dedicated to aggregating sensor data. Understanding the landscape of LLM research and the scope of sensor data aggregation through existing web portals provides a foundational understanding for the proposed methodology in this study. Finally, previous work related to the transformation of sensor data into structured formats is described, underscoring the contributions of this study to the state-of-the-art.

### 2.1. Large Language Models

In recent years, natural language processing (NLP) has experienced a transformative evolution with the emergence of LLMs. The primary function of a language model is to predict and generate coherent language. These models evaluate the likelihood of individual tokens or sequences of tokens within a broader context. Predicting subsequent elements in a sequence is essential across various tasks, such as text generation [6], language translation [7], and question answering [8], among others.

LLMs have a significant number of parameters, ranging from hundreds of millions to billions. This scale empowers them to capture intricate linguistic patterns, harness contextual cues, and discern nuanced semantic meanings that previously posed challenges for NLP systems. Constructing LLMs demands a complex and resource-intensive process. As models expand in size, they concurrently augment in complexity and effectiveness. These larger-scale language models are often pre-trained on extensive textual corpora, enabling them to acquire broad language patterns and world knowledge. Subsequent to pre-training, these models can undergo fine-tuning for specific NLP tasks, rendering them versatile tools adaptable for a variety of applications.

Earlier models could predict single words, whereas modern LLMs can forecast probabilities for entire sentences, paragraphs, or even whole documents. The rapid expansion of language models in recent years is driven by increased computer memory, larger datasets, enhanced processing power, and more effective techniques for handling longer text sequences.

A key advancement in language models emerged in 2017 with the introduction of Transformers [9], an architecture built around the concept of attention. This innovation unlocked the ability to handle longer sequences by concentrating on the most salient components of the input, effectively resolving memory constraints that earlier models grappled with. Transformers comprise two primary components: an encoder and a decoder. The encoder converts input text into an intermediate representation, while the decoder translates this intermediary representation into coherent text output. Transformers have established themselves as the cutting-edge architectural framework for a diverse array of language model applications.

The rapid advancements in LLMs have witnessed groundbreaking progress in both research and industrial applications. In 2018, the advent of the Bidirectional Encoder Representations from Transformers (BERT) [10] revolutionised the comprehension and generation of human languages. Unlike its predecessors that processed text in a unidirectional manner, BERT introduced bidirectional learning. It simultaneously considered both left and right contexts when training on a word, enabling a comprehensive understanding of its entire context. This bidirectional comprehension empowered BERT to capture intricate linguistic relationships and nuances, leading to state-of-the-art performance across a diverse array of NLP tasks. BERT’s success spanned applications such as sentiment analysis [11], text classification [12], machine translation [13], and question answering [14].

The influence of BERT on NLP research and applications has been profound, fostering the development of more advanced LLMs. It has paved the way for a new era in natural language comprehension and generation, with its techniques adopted and expanded upon in various other models [15]. For instance, GPT-3 [16], boasting 175 billion parameters, demonstrates the capability to generate text and code based on concise written prompts. Another example, Megatron-Turing [17], among the world’s largest models with 530 billion parameters, specialises in reading comprehension and natural language inference, facilitating tasks like summarisation and content generation. Equally noteworthy is BLOOM [18], an open LLM proficient in generating text across 46 natural languages and more than a dozen programming languages.

A recent addition to this landscape is ChatGPT (https://chat.openai.com/, accessed on 18 September 2023), emerging as a state-of-the-art LLM. Developed by OpenAI (https://openai.com/, accessed on 18 September 2023), this model, built upon the GPT-3 architecture, excels in generating human-like texts and engaging in multilingual conversations. ChatGPT is specifically fine-tuned to excel in producing conversational responses. It adeptly furnishes coherent and contextually relevant answers to a diverse array of questions and prompts. Much like its precursor GPT-3, ChatGPT showcases robust contextual comprehension, retaining context throughout extended conversations and delivering context-aware responses based on previous messages. Users interact with ChatGPT by presenting prompts or inquiries. ChatGPT can process prompts encompassing both images and text, rendering it a versatile tool adaptable for a multitude of tasks.

Finally, Llama 2 [19], an open-source LLM introduced by Meta AI (https://ai.meta.com/, accessed on 18 September 2023), represents a versatile tool applicable across various domains. It encompasses capabilities including text generation, language translation, and the production of diverse creative content. Additionally, Meta AI introduced Code Llama [20], tailored specifically for code-related tasks. These models showcase cutting-edge performance, support for extensive input contexts, and even possess zero-shot instruction-following abilities for programming tasks. Llama 2 is available in three sizes, delineated by the number of parameters: 7 billion, 13 billion, and 70 billion.

### 2.2. IoT Portals

In the realm of IoT device exploration, various platforms have emerged to streamline the discovery and accessibility of sensors. Shodan (https://www.shodan.io/, accessed on 18 September 2023), an online search engine and scanning service, specialises in detecting and monitoring Internet-connected devices and systems. Conceived by John Matherly and launched in 2009, this search engine scans both IPv4 and IPv6 spaces [21]. Shodan is an interesting option for locating IoT devices owing to its extensive database of devices and online services, making it a valuable resource for research and analysis. Moreover, it offers a public API, enabling developers to programmatically access and leverage Shodan’s data, facilitating research and custom development [22]. Additionally, it features data visualisation tools that streamline comprehension and analysis of the gathered information. However, the free version of Shodan imposes limitations on the number of search results, potentially impeding extensive research efforts. It is noteworthy that Shodan does not interpret sensor outputs in terms of deciphering analogue or digital data from specific sensors. Its primary function revolves around indexing and presenting information regarding devices connected to the network.

Censys (https://search.censys.io/, accessed on 18 September 2023), an online search engine and scanning service akin to Shodan, specialises in tracking and compiling information about devices and resources on the Internet. It is open source and freely available for academic purposes [21]. This platform enables users to conduct advanced searches to locate devices and services based on diverse criteria. Similar to Shodan, Censys maintains an expansive database of Internet-connected devices and services, alongside offering a well-documented public API. Utilising passive scanning techniques, Censys gathers information without disrupting the regular functioning of the scanned systems. Nevertheless, Censys presents certain limitations. Its data are typically provided in raw format, requiring users to possess technical proficiency for effective interpretation. Reports from Censys encompass information on services, protocols, and SSL/TLS certificates, among others, necessitating a level of technical understanding for accurate analysis. Moreover, user interaction with Censys may require account creation after surpassing specific usage thresholds, and API access is rate-limited by token buckets [23].

ZoomEye (https://www.zoomeye.org/, accessed on 18 September 2023) serves as an online search and scanning platform designed to pinpoint and monitor devices and systems connected to the Internet. Much like Shodan and Censys, ZoomEye specialises in gathering information about online assets, encompassing servers, cameras, routers, IoT devices, and various other Internet resources. It provides users with diverse filtering criteria for refining their searches and offers an accessible API. However, a notable drawback is that this search engine does not support general searches across all devices, since users must know thee specific keywords to conduct searches.

### 2.3. Transforming Sensor Data to Structured Formats

Transforming sensor data into structured formats is a crucial step in rendering the data usable for various applications. This section reviews key research contributions that have advanced methods and technologies for this transformation process.

New transformer models have been developed, such as the Soft Sensing Transformer Model [24], which aims to transform sensor readings into structured formats in the same way that sentences are structured in natural language, thus providing a novel method to handle sensor data in industrial settings. Although this model demonstrates the potential of machine learning techniques in interpreting and structuring sensor data, they do not consider end users and their information needs.

With respect to structured sensor data formats, the heterogeneity of data formats in IoT is addressed in [25]. This study proposes converting raw sensor data into the Sensor Markup Language (SenML) and transforming it into a semantic representation based on RDF. Another noteworthy contribution [26] introduces a novel script language, Language for Sensor Data Description (L4SDD), to achieve cross-domain syntactic interoperability. L4SDD focuses on defining a unified output data format and converting sensor data into this format, thus facilitating the integration of sensor data from various domains. The primary limitation of these approaches is the requirement for new languages to be applied to sensor data, which impedes automation.

In the application of LLMs to sensor data, several approaches focus on specific scenarios. For instance, the use of LLMs for forecasting traffic accidents from sensor data are explored in [27], while integrating LLMs into multiagent autonomous systems to generate GPT-compatible prompts for unifying collected sensor data are described in [28]. However, these approaches are limited to specific domains and do not address broader scenarios to enhance the reusability of sensor data.

These studies collectively contribute to the methodologies for transforming sensor data into structured and standardised formats. Such transformation is vital for advancing IoT, industrial automation, and data analytics. Yet, existing work often overlooks data reusers, lacks automation, or is limited to domain-specific scenarios. To address these gaps, the present work provides a methodology leveraging LLMs to convert raw sensor data into structured formats. This methodology considers the objectives of data reusers, achieves a high level of automation, and is applicable across various domains.

## 3. Methodology

As mentioned in Section 1, this study introduces a methodology aiming to convert sensor data and metadata into a more interpretable and reusable format. As stated before, handling sensor information poses challenges due to system and data heterogeneity, demanding substantial effort for effective management. The proposed methodology seeks to enhance sensor data accessibility by leveraging contextual understanding and text generation capabilities inherent in modern LLMs to convert raw sensor data into a reusable format.

**Figure 1:**
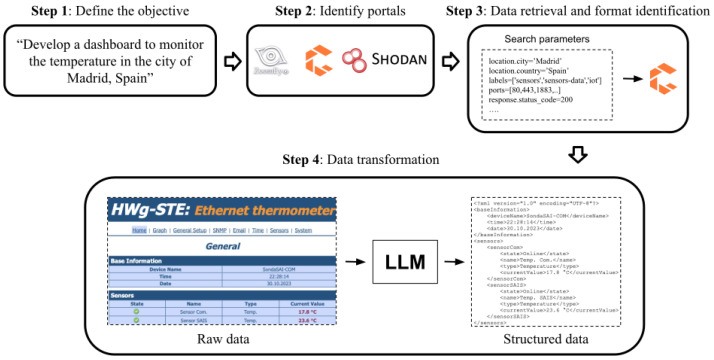
Four steps in the proposed methodology for obtaining structured information from raw sensor data.

The methodology involves several steps: defining objectives, identifying sensor data portals, filtering sensors based on requirements, retrieving data, and utilising a LLM to parse raw sensor data, transforming it into a structured format suitable for exchange and reuse. Figure 1 provides a visual overview of this process.

***Step 1**:*  *Define the objective*

Before embarking on any project, the initial step entails thorough contemplation and a clear conceptualisation of the tasks at hand. It is pivotal to identify the essential data for achieving the desired outcomes and to choose a compatible output format for subsequent utilisation. This preliminary phase demands significant time and effort as it lays the groundwork for subsequent steps.

***Step 2**:*  *Identify portals*

The second step initiates with the identification of portals potentially beneficial for the objectives set in the prior phase. As highlighted in Section 2, different portals are available for IoT device exploration. These portals serve as valuable resources for discovering data sources aligned with the methodology proposed. Each provides tools, including APIs enabling programmable queries to their databases and filtering capabilities adaptable to specific needs.

However, as previously noted, access limitations such as monthly API call quotas or constraints on the number of search filters exist. Additionally, some portals exhibit greater coverage in particular geographical regions (e.g., ZoomEye in China). Therefore, it is imperative to ascertain that the chosen portal aligns with the required data and fulfils the criteria established in the initial phase of the methodology.

***Step 3**:*  *Data retrieval and format identification*

The third step involves querying sensors within the selected portals by employing specific search parameters to extract pertinent information. Various filters enable the search for sensors with distinct features. For instance, in the case of the Censys portal, filters such as “tag:IoT” or “tag:Sensor” aid in sensor identification. To refine the search within a specific geographic area, filters like “location.city: Madrid, Spain” can be applied. Filters’ definitions differ across portals, necessitating consideration of each one’s specific syntax. However, it is crucial to note that all portals share a common syntax foundation.

Significant filters pertain to the services enabling access to sensor data. For instance, filtering results within the Censys portal using the aforementioned labels reveals that a majority of sensors have HTTP or MQTT (https://mqtt.org/, accessed on 18 September 2023) services enabled. The frequently open ports are 1883 (typically used by the MQTT protocol) and 80. Additionally, filtering for a response status of 200 (indicating a successful response code) using “response.status” is crucial to avoid defunct or malfunctioning sensors. By employing these filters, the search engine retrieves results wherein sensor data are primarily shared in HTML format combined with XML or JSON responses.

***Step 4**:*  *Data transformation*

The final step of the methodology involves employing a LLM to parse raw sensor data, converting it into coherent structured information. This process necessitates defining a comprehensive prompt outlining the specific data required (e.g., meteorological information) and specifying the preferred output format (e.g., XML or JSON).

Consequently, this prompt facilitates the parsing of sensor data delivered in formats like HTML, illustrated in Listing 1. The raw data can be transformed by a LLM into a more usable format, such as JSON, ensuring proper content structuring for reusability. The resulting output, exemplified in Listing 2, yields data in a more reusable format, aligned with the FAIR principles [29].

**Listing 1.** Raw data from sensor in HTML format.

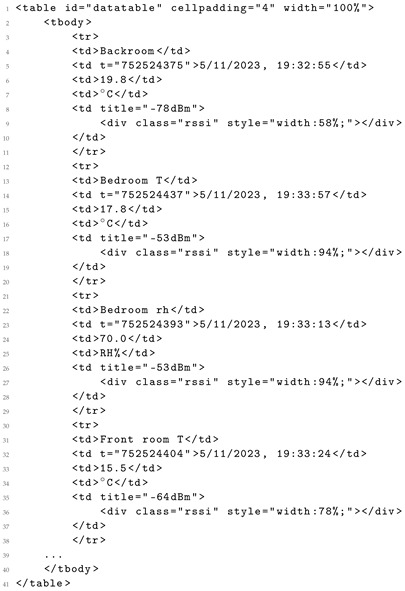



**Listing 2.** Equivalent JSON generated by a LLM.

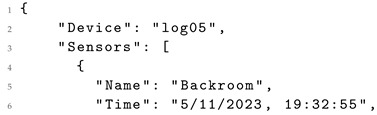



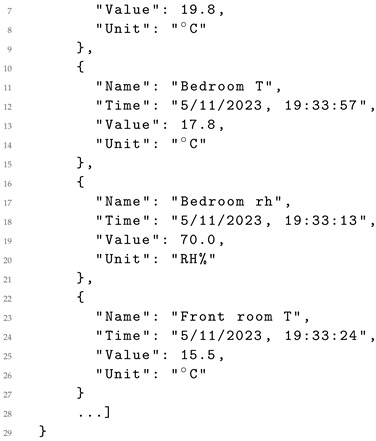



## 4. Use Case

This section encapsulates the application of the proposed methodology in a specific use case, demonstrating its practical implementation. It showcases the step-by-step execution of the methodology, highlighting its real-world applicability and effectiveness in handling concrete scenarios.

***Step 1**:*  *Define the objective*

The primary goal of the proposed use case revolves around retrieving meteorological data from sensors. As stated in the methodology, the aim is to transform these data, if initially presented in an inaccessible format, into a user-friendly structure using a LLM. Consequently, the focus of the use case is narrowed to meteorology and forecasts data.

***Step 2**:*  *Identify portals*

Initially, the selection of the IoT device information source is imperative. Initially, a comprehensive search was conducted to identify existing IoT portals in the market. The selection focused on those offering the most extensive content. Consequently, the primary portals chosen for this study, as detailed in Section 2, were Censys, Shodan, and ZoomEye. After evaluating these portals, Censys emerged as the chosen platform. Several factors contributed to this decision:

1. Extensive API documentation and diverse filter options;
2. Rapid search capabilities;
3. Extensive device database;
4. Intuitive user interface;
5. Cost-effectiveness.

***Step 3**:*  *Data retrieval and format identification*

Next, the data retrieval process from the selected Censys portal is essential. It is important to highlight that raw data were specifically sought to showcase the capabilities of LLMs. The decision was made to focus on HTML format, as it is commonly used by most sensors in the portals analysed. Specifically, information about sensor formats accessible through HTTP with a response status of 200 is sought. This subset constitutes 46.71% of the sensors documented in Censys. Regrettably, the remaining 53.29% do not provide discernible format information—either they don not operate with HTTP, or yield no valid response. Among the viable subset, the breakdown of IoT device format percentages is as follows: 82.9% exclusively provide information in HTML, 0.25% solely furnish details in XML/JSON, 11.72% offer both HTML and XML/JSON information, while the residual 5.13% present data in other formats. Notably, the small proportion of sensors returning information in XML/JSON aligns with the objectives of this research proposal, highlighting the necessity for the proposed methodology to transform data into reusable formats.

Adhering to the proposed methodology, the following steps outline the process for data retrieval:

1. User poses a query.
2. The query, incorporating pertinent filters, is sent to the Censys API.
3. A set of endpoints/URLs facilitating access to various sensors is obtained.
4. Information from these sensors is retrieved.

Listing 3 provides an example showcasing sensor output retrieved from Censys, tailored for an average user profile seeking current-day information on temperature and humidity, among others, in Port Louis (Mauritius).

**Listing 3.** Data retrieved from Censys in JSON format for an average user profile.

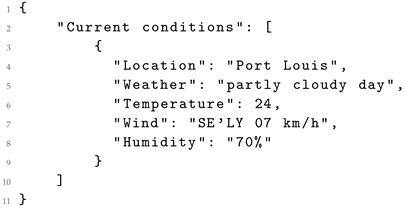

***Step 4**:*  *Data transformation*

As detailed in Section 3, an LLM is employed to converting the raw sensor information into a more accessible format. Selecting an LLM involves considering various factors to ensure alignment with the specific needs and objectives of the task. Key aspects include precision and recall, which assess the model’s capability to generate structured data from raw sensor data. These metrics are evaluated for different models in Section 5 of this work. Customisation possibilities of the model, such as the ability to be fine-tuned for specific tasks or domains, are also crucial. Security and privacy considerations are paramount. It is necessary to evaluate the model’s security features, particularly its handling of sensitive data, to ensure compliance with applicable privacy laws and regulations.

Latency and response time are vital, especially if the LLM is to be used in customer-facing applications where quick responses are essential. Scalability is another critical factor, ensuring that the model can handle the anticipated volume of requests and scale up as system demand increases. The cost and licensing of the LLM are also relevant; some models are free, while others incur usage fees. It is imperative to ensure that licensing terms align with the intended use of the technology.

From a more technical standpoint, the support and documentation offered by the LLM are important. Models with comprehensive documentation and reliable technical support are preferable. Additionally, the developer and user community surrounding the model should be considered, as a robust community can provide valuable support and drive innovation.

**Figure 2:**
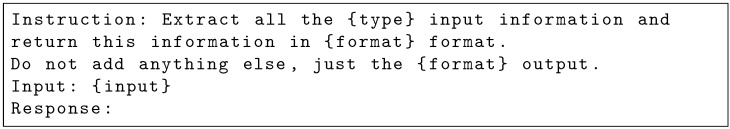
Prompt template to transform raw input data of a selected type into a specified structured output format.

Defining an accurate prompt for the language model is crucial to attain the desired objective. In this use case, the prompt depicted in Figure 2 provides the extraction of pertinent information from the input it receives, facilitating the transformation into a structured format. For instance, in this prompt, if the content in Listing 1 was used as the {input} parameter, the Response generated by the LLM would be as shown in Listing 2.

The initial segment of the prompt delineates the directives for the model to accomplish the proposed objective, providing instructions regarding the desired output format. This ensures the generation of data by the LLM in the specified format. Subsequently, the prompt includes unprocessed sensor data (input). The final line (Response) serves as an indicator that the following text generated by the model will constitute the response. The roles of the variables within the preceding prompt are described below:

- type: represents the category of data provided by the sensor (e.g., “meteorology”).
- format: specifies the desired format for the model’s output transformation (e.g., “XML”).
- input: denotes the content of the input to be transformed (raw sensor data).

When the input to the model exceeds the model’s input length, it is necessary to split the input into smaller parts for processing. Subsequently, after processing each segment, the results are merged into a single file.

## 5. Evaluation

This section focuses into the testing phase of the use case proposed earlier, encompassing three distinct types of tests: evaluating model performance by assessing precision and recall in transforming raw sensor data into structured formats, analysing the computational time required for data transformation, and conducting qualitative assessments by manually reviewing XML/JSON generated by the models from raw sensor data. Each of these is described in more detail below.

### 5.1. Dataset

Before describing the evaluation conducted, this section will first describe the dataset used in the experiments. To test the performance of the LLMs in the proposed use case, 25 sensor accesses have been compiled from Censys, each of them offering data in both HTML and XML/JSON formats. These sensors were selected from a subset that provides data in both formats, facilitating a comparative analysis of the resulting outputs generated by the LLMs. It is important to emphasise that the information provided in Censys by a sensor in HTML and XML/JSON formats may not always align. A manual examination of the dataset revealed that XML/JSON data often encompasses additional fields not found in the corresponding HTML file. Given that LLMs utilise HTML content to generate structured output, the comparison between this output and the original XML/JSON format from the sensor may result in lower performance. This discrepancy is not indicative of an error in the LLM’s output generation; rather, it stems from the absence of attributes in the HTML source that are present in the XML/JSON data.

Table 1 offers an overview of the collected dataset for both HTML and XML/JSON data. This dataset, which provides data in both HTML and XML/JSON formats, was obtained using the query stated in Listing 4 in Censys.
**Listing 4.** Query that obtains data from Censys.(labels:sensors OR labels:IoT OR labels:sensors-data) and(services.http.response.body: "json" ORservices.http.response.body: "xml") andservices.http.response.body: "html" ANDservices.http.response.status\_code=200

### 5.2. Model Performance

The primary objective of this evaluation is to assess the quality of XML/JSON generated by the LLMs from HTML data. The comparative analysis involves measuring the model’s precision (indicating how many values in the generated file correspond to the original HTML and XML/JSON formats) and the model’s recall (denoting how many values in the original HTML and XML/JSON are present in the generated file). Subsequently, the F-score is calculated for both the original HTML and XML/JSON, representing the harmonic mean of precision and recall.

Each of the sensors provides varying meteorological data for diverse global locations. Some sensors offer a substantial volume of information to structure, while others provide more limited data. The available data spans from current temperature and humidity to comprehensive weather forecasts. Evaluating the different types of data from these sensors is pivotal to observe how diverse models interpret and handle them.

For this evaluation, the selected LLMs included *Llama 2 7B* (https://huggingface.co/meta-llama/Llama-2-7b-chat-hf, accessed on 20 October 2023) (the version with 7 billion parameters) and *Llama 2 13B* (https://huggingface.co/meta-llama/Llama-2-13b-chat-hf, accessed on 20 October 2023) (13 billions parameters), from Meta, alongside *GPT-3.5 Turbo* and *GPT-4* from OpenAI. These choices were made based on specific criteria. OpenAI models stand as established benchmarks in the field, while Meta models, being close competitors, are freely accessible. The *Llama 2 70B* (https://huggingface.co/meta-llama/Llama-2-70b-chat-hf, accessed on 20 October 2023) (70 billions parameters) model was also tested, but it was discarded since it was resource-intensive and the initial results did not justify their inclusion in the evaluation.

Integration of these LLMs was facilitated through *LangChain* (https://www.langchain.com/, accessed on 20 October 2023), a framework engineered to streamline LLM application development. *LangChain* offers versatility across diverse use cases, including the extraction of structured information from text, as applied in this methodology.

**Figure 3:**
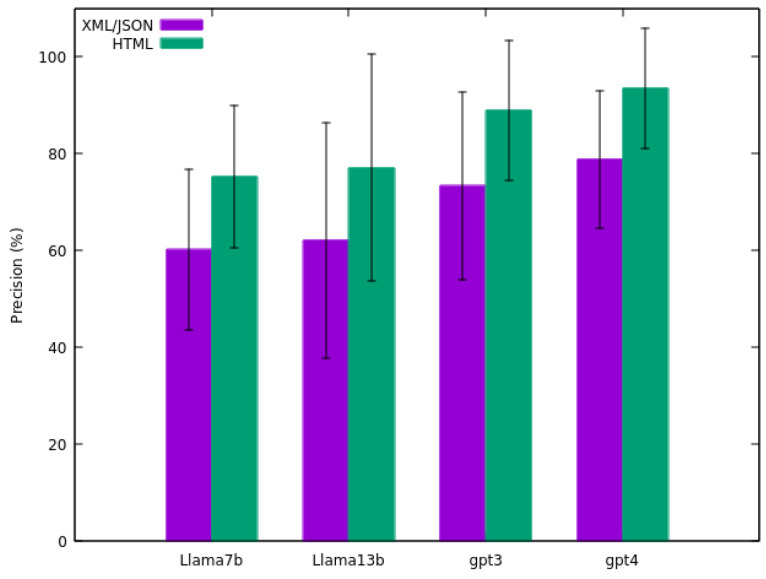
Average precision of the four models tested on the sensors dataset.

Figure 3 illustrates the results of the precision calculation (the average of the precision results of the 25 sensors) alongside the standard deviation for each model. The comparison presented is the outcome of matching fields between the original files (XML/JSON and HTML) and the file generated by the LLM. For the HTML comparison, fields were manually checked to evaluate the alignment.

Observing the results, the *GPT-4* model demonstrates the highest precision (78.74% for comparison with XML/JSON and 93.51% for comparison with HTML), while the *Llama 2 7B* model exhibits the lowest precision (60.24% for comparison with XML/JSON and 75.33% for comparison with HTML). It is noteworthy that comparisons yield better results for HTML than for XML/JSON. As mentioned earlier in this section, this discrepancy arises because the original XML/JSON might contain data not included in the corresponding HTML version, hence is unextractable by the models.

Additionally, substantial deviation is evident in the results. For instance, the *GPT-4* model exhibits a notable deviation (78.74 ± 14.24% for comparison with XML/JSON and 93.51 ± 12.43% for comparison with HTML). This deviation implies significant variability within the model’s performance across different sensor data.

**Figure 4:**
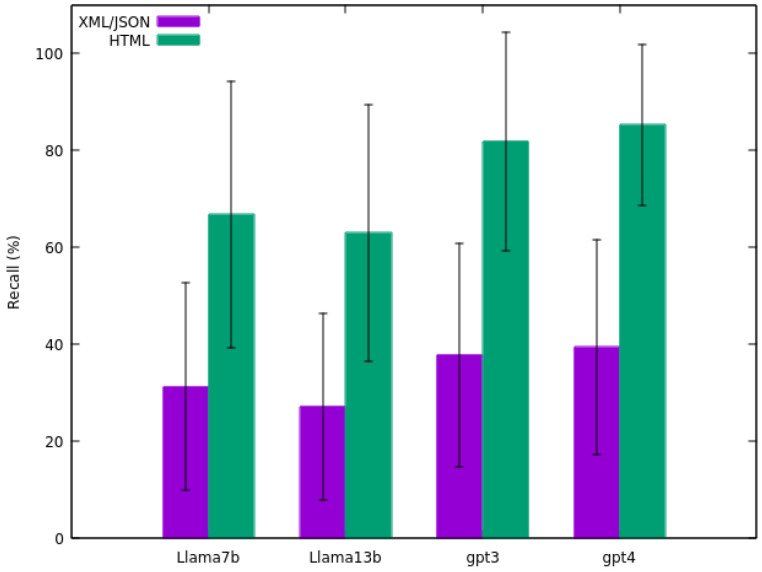
Average recall of the four models tested on the sensors dataset.

From Figure 4, the recall calculation results are presented, showcasing the average recall outcomes for the 25 sensors. A significant disparity is evident between comparisons with original HTML and XML/JSON files. For instance, 37.78% for comparison with XML/JSON and 81.87% for comparison with HTML for the *GPT-3.5 Turbo* model. As mentioned previously, this divergence is due to the nature of the XML/JSON data from the sensor, which may contain information not present in the original HTML content used by the model for extraction. Consequently, this inaccessible information can not be reflected in the output generated by the LLM, leading to a reduced recall when comparing with XML/JSON, as the model generates information solely from HTML content.

It is noteworthy that, once again, the *GPT-4* model exhibits the highest recall (39.51% for comparison with XML/JSON and 85.33% for comparison with HTML). Conversely, this time, the *Llama 2 13B* model yields the least favourable results (27.24% for comparison with XML/JSON and 62.98% for comparison with HTML). Similar to the precision findings, this numbers also reflects considerable deviation in the results (e.g., 39.51 ± 22.16% for comparison with XML/JSON and 85.33 ± 16.65% for comparison with HTML for the *GPT-4* model).

**Figure 5:**
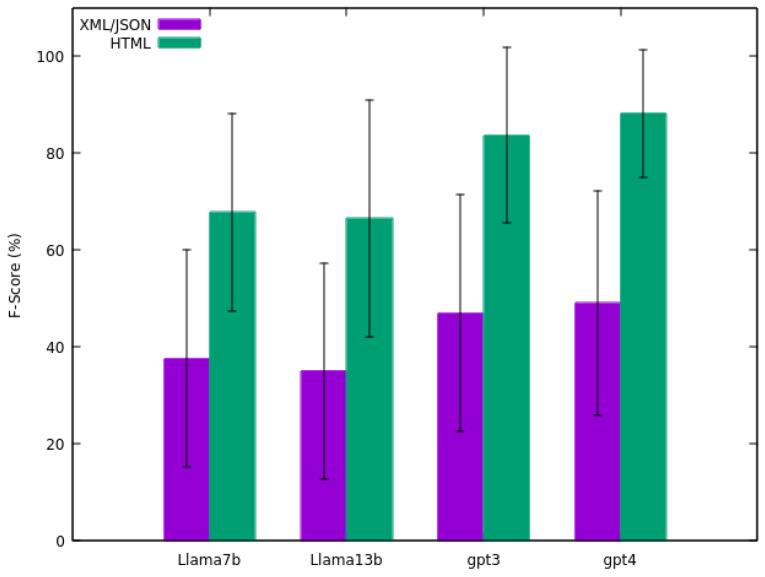
Average F-score of the four models tested on the sensors dataset.

Finally, the F-score results are illustrated in Figure 5. Notably, the OpenAI models demonstrate the most promising overall outcomes. *GPT-3.5 Turbo* model achieves 46.96% for comparison with XML/JSON and 83.69% for comparison with HTML, while the *GPT-4* model attains 49.13% for comparison with XML/JSON and 88.21% for comparison with HTML. Once again, the results exhibit substantial variance (e.g., 46.96 ± 24.54% for comparison with XML/JSON and 83.69 ± 18.10% for comparison with HTML for the *GPT-3.5 Turbo* model).

### 5.3. Execution Time

During the assessment of model precision and recall, the time taken by each model to perform the format transformation of a given input was recorded. The Meta models were executed on a server equipped with an NVIDIA A100-SXM4-40 GB GPU (manufactured by NVIDIA Corporation, Santa Clara, CA, USA), an AMD EPYC 7742 64-Core Processor CPU (manufactured by Advanced Micro Devices Inc., Santa Clara, CA, USA), and 1 TB RAM. Conversely, the OpenAI models operated on the OpenAI servers, which, while undisclosed in terms of hardware specifications, are evidently more robust than the server hosting the Meta models, leading to a substantial disparity in execution times.

**Figure 6:**
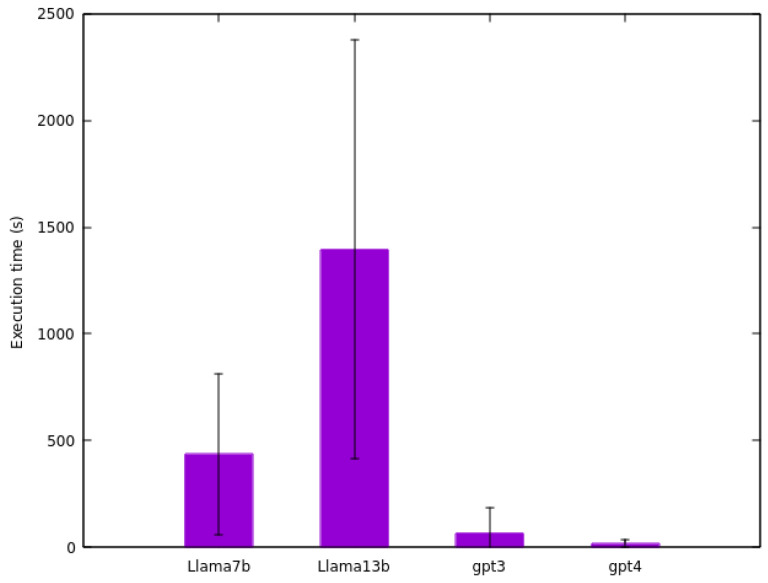
Average execution time (in seconds) of the four models on the sensors dataset.

Figure 6 shows the time consumption for each model in transforming content from HTML to structured format. Notably, the *Llama 2 13B* model exhibits the lengthiest average transformation time among the four models (1395.75 s), due to its intensive computational demands. Remarkably, a particular sensor’s data transformation spanned 4.534 s for this model. The input file in this case comprised 309 lines. Additionally, it is observed that the *GPT-3.5 Turbo* model (68.43 s) consumed more time compared to *GPT-4* (18.13 s), potentially owing to API demand fluctuations inherent to the GPT models.

### 5.4. Qualitative Assessment

Following the experiments, a qualitative assessment of the structured data generated by the different models was conducted using a sample of 10 sensors randomly selected from the total 25. This manual inspection aims to provide a qualitative evaluation of the results.

As an example, consider the assessment of one such sensor. The original XML data (Listing 5) displays certain elements such as “Normal” or “0” that are not present in its corresponding HTML version (Listing 6). Consequently, these elements are unattainable for the LLMs to generate in the resultant XML (Listing 7), in this case using *GPT-4*. This example elucidates the rationale behind comparing both HTML and XML/JSON data as performed in the earlier analysis.

**Listing 5.** Original XML data from the sensor.

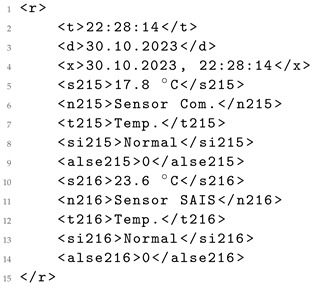



**Listing 6.** Content from the original HTML data from the sensor.

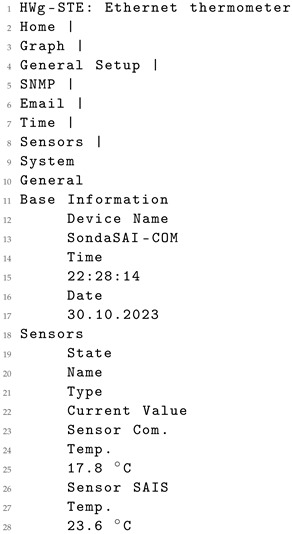



**Listing 7.** XML generated from the original HTML data using GPT-4.

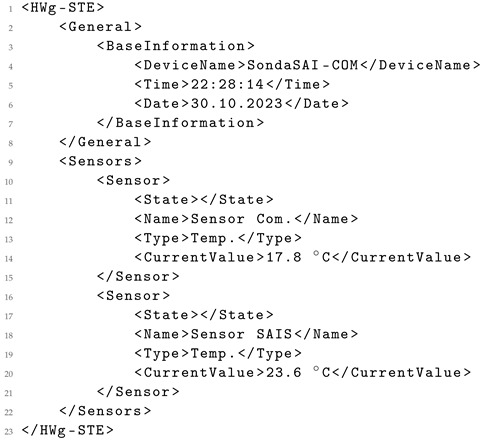



The structured information in the generated XML shown demonstrates clear and intuitive formatting, distinct from the original XML, which lacks this level of clarity. Additionally, the naming of elements in the generated XML (Listing 7) is notably descriptive and accurate when compared to the original XML (Listing 5).

However, it is important to highlight instances of model hallucination observed in the analysis. For instance, Listing 8 illustrates this phenomenon in the output generated by *Llama 2 7B*, where values such as “Temp.”, “Sensor Com.”, and “Sensor SAIS” from the HTML content (Listing 6) are renamed by the model to “Temperature”, “Temp. Com.”, and “Temp. SAIS”, respectively. Another example is the generation of the “Online” value for sensor status, seemingly without a clear basis or context for assigning this value. It is worth noting that efforts are underway to address these hallucination issues in model enhancements [30].

**Listing 8.** XML generated from the original HTML data using Llama 2 7B.

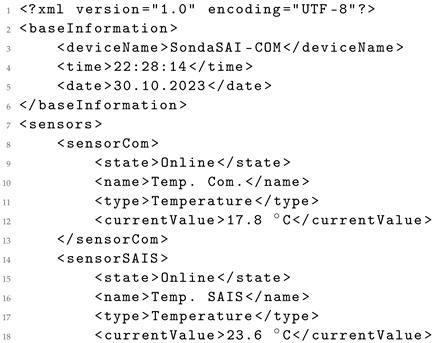



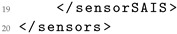



It is important to highlight the potential variability in structure observed in the generated data. While a standard structure is predominantly maintained, there have been instances where certain values are included as attributes of an element, as exemplified in Listing 9 generated by *Llama 2 13B*. Specifically, the sensor name, type, and current_value are attributes within the sensor element, differing from independent elements shown in the original XML in Listing 5. These structural variations can be adjusted in the prompt (Figure 2) by indicating a preference for presenting data as attributes or as independent elements.

**Listing 9.** XML generated from the original HTML data using Llama 2 13B.

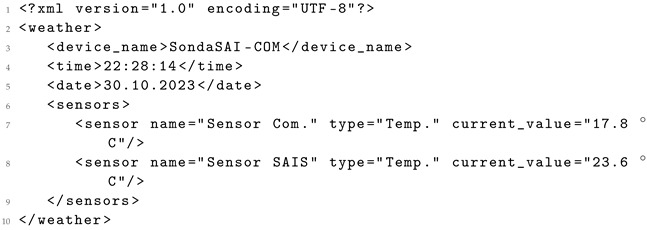



## 6. Discussion

This section discusses the findings from the evaluation presented in Section 5. The evaluation demonstrated the capabilities of these models in accurately and effectively converting data from HTML to XML/JSON formats, with *GPT-4* showing particularly high precision and recall rates.

The aim of this study was to develop a methodology for enhancing the reusability and interoperability of sensor data. The results indicate that *GPT-4* achieved the highest precision at 93.51% and recall at 85.33%, closely followed by *GPT-3.5 Turbo* and outperforming other models like *Llama 2 7B* and *Llama 2 13B*. This suggests that advanced LLMs like *GPT-4* are highly effective in parsing and restructuring complex data formats. The performance of these models is indicative of their potential in enhancing data reusability and interoperability within IoT infrastructures, a key objective of this study. These findings guarantee the high efficacy of the methodology in transforming raw sensor data into structured formats, thus fulfilling the primary objective of this work.

Furthermore, *GPT-4* not only excelled in accuracy, but also in time efficiency, emerging as the fastest among the four models in executing the transformations. This efficiency is noteworthy, considering the complexity of the tasks involved. On the other hand, *Llama 2 13B*, despite being a more complex and presumably more capable model, was the least effective in the experiment and also the slowest. This underperformance is particularly striking when compared to the smaller *Llama 2 7B* model. The results indicate that larger, more complex models do not necessarily guarantee better performance in data transformation tasks, especially when considering time efficiency as a critical factor.

The unexpected underperformance of *Llama 2 13B*, compared to its lighter counterpart, raises questions about the efficiency-accuracy trade-offs in model selection for specific tasks. While larger models are generally expected to perform better, their increased computational requirements can lead to longer processing times and diminished practicality, especially in time-sensitive applications. This finding suggests a need for a more nuanced approach to selecting models based on a balance between accuracy, processing time, and computational resource requirements.

The evaluation of the models not only highlighted *GPT-4*’s superior performance in terms of accuracy and time efficiency, but also revealed important insights into the relationship between model size, complexity, and effectiveness in data transformation tasks. These insights will be valuable for future research and practical applications involving large language models in data processing.

A notable aspect of the results is the variability in model performance across different sensor data types, as evidenced by the standard deviation values presented in Figure 3, Figure 4 and Figure 5. Even the top-performing model, *GPT-4*, exhibits a standard deviation of 12.43 in precision, 16.65 in recall, and 13.51 in F-score. This variability highlights the challenges in achieving consistent data transformation results in real-world scenarios. The observed differences in precision and recall across models also underscore the need for careful selection of models based on the specific requirements of the data transformation task.

In Section 5, the models have been tested in terms of precision, recall, and F-score. Additionally, it is interesting to analyse how these LLMs compare to traditional data conversion techniques. Various methods and approaches exist for converting, manipulating, and optimising data:

- *Schema conversion*: converts data from one logical structure to another, adapting from formats like relational to non-relational, hierarchical to flat, or normalised to denormalised.
- *Format conversion*: transforms data from one physical representation to another, such as from binary to text, JSON to XML, or compressed to uncompressed.
- *Type conversion*: changes data from one type to another, like string to integer, float to decimal, or boolean to bit.
- *Encoding conversion*: modifies data from one character set to another, for example, ASCII to UTF-8, or ISO-8859-1 to UTF-16.
- *Date and time conversion*: reformats dates and times for consistency, such as changing 2023-09-21 to 09/21/2023.
- *Number conversion*: alters the representation of numbers, like converting an integer to a decimal (e.g., 5 to 5.0), significant for precise calculations.

From these traditional methods, LLMs are capable of performing schema conversion, as they have shown proficiency in programming tasks [20]. They can also execute format conversion, demonstrated in this study where HTML format was converted to JSON and XML. Furthermore, models like *GPT-4* have demonstrated the ability to compress and uncompress files. They can also perform type conversion, dealing with string formats and numerical tasks [31], as well as encoding conversion, date and time conversion, and number conversion, handling various text formats.

Therefore, the capabilities of these models in the realm of data conversion are vast, covering traditional techniques previously implemented using separate tools, such as Apache Sqoop (https://sqoop.apache.org/, accessed on 13 November 2023) for schema conversion and Apache Avro (https://avro.apache.org/, accessed on 13 November 2023) for format conversion, but now unified in a one-stop solution offering a more flexible and efficient proposal. The ability of these models to handle various types of data conversion positions them as valuable tools in diverse data processing workflows.

## 7. Conclusions and Future Work

This study detailed the challenges faced in harnessing the potential of sensor-generated data within IoT infrastructures, proposing a methodology aimed at overcoming barriers to data reusability and accessibility. Despite the immense volume of data generated by IoT infrastructures, limitations in data handling, proprietary formats, and interoperability challenges hinder its effective utilisation by third parties. The European Data Act initiative endeavours to regulate and enhance access to sensor-generated data, aiming to ensure accuracy, completeness, relevance, and intellectual property rights. However, challenges persist in achieving technical interoperability, primarily due to heterogeneous data and disparate sensor system architectures.

The methodology proposed in this paper aims to leverage LLMs to convert raw sensor data (e.g., in HTML format) into standardised structured formats (e.g., JSON or XML), aiming to facilitate data reuse and comprehension. The outlined methodology involves defining data requirements, identifying data sources, retrieving data, and utilising LLMs to transform raw sensor data into structured, reusable formats. A use case focusing on meteorological data retrieval from a sensor web portal was employed to test this methodology, with an evaluation emphasising precision tests of various LLMs for transforming HTML sensor data into XML format.

The assessment of LLMs revealed that *GPT-4* achieved the highest precision in transforming HTML to structured format, reaching 93.51%. Conversely, the lowest precision was observed with *Llama 2 7B* at 75.33%. Similarly, *GPT-4* demonstrated the best recall, achieving 85.33%, while *Llama 2 13B* had the lowest recall at 62.98%. These results underscore the high performance of LLMs, rendering them well-suited for the task proposed.

In terms of execution time, OpenAI models (*GPT-3.5 Turbo* and *GPT-4*) notably outperformed Meta models. However, direct comparison is complex due to different hardware configurations for running these models.

An interesting point to note is that while OpenAI models involve a subscription fee, Meta models are freely available and were executed on a personal server without added cost. In a production environment, these factors, alongside performance metrics, should be considered carefully.

The manual review of structured outputs revealed both the strengths and limitations of LLMs. Notably, the clarity and descriptive accuracy of the generated XML, contrasting the original XML, highlighted the language models’ ability to enhance data presentation. Yet, instances of hallucination, wherein models incorrectly renamed data elements, were identified. Addressing such issues remains a significant area for model improvement.

A critical area for future exploration involves the examination of alternative methodologies or frameworks dedicated to converting raw sensor data into structured, reusable formats. Additionally, assessing the practical implementation and scalability of LLMs in transforming HTML-based sensor data into standardised formats remains a crucial focal point. This endeavour necessitates a meticulous analysis of potential challenges, computational requisites, and practical considerations involved in harnessing LLMs. Understanding these aspects is integral to leveraging LLMs effectively for the transformation of sensor data.

To enhance the outcomes of the experiments, an instruction fine-tuning process [32] is planned for implementation in the future. In this process, LLMs are tailored specifically for the task of data transformation based on explicit instructions. This approach, extending beyond traditional fine-tuning, incorporates high-level instructions or demonstrations to guide the model’s behaviour. The process will leverage sensor data from open data portals, which typically provide datasets in various formats such as JSON, HTML, and CSV. These data have to be preprocessed to furnish the model with the necessary instructions, as shown in Figure 2, with the input dataset in HTML format and the desired output in XML/JSON format.

Furthermore, investigating various use case scenarios and user profiles for sensor data utilisation presents a significant opportunity. This entails a detailed examination of specific needs, requirements, and challenges across diverse applications. Understanding these unique needs is pivotal in tailoring data retrieval and transformation methods to suit varied end-user requirements.

Lastly, conducting a comprehensive evaluation of different language models in transforming sensor data into structured formats remains a vital pursuit. This involves extensive testing, model comparisons, and exploration of challenges such as model hallucination and variations in output structures.

## Figures and Tables

**Table 1:** Characteristics of the collected HTML and XML/JSON datasets.

Characteristic	HTML	XML/JSON
Number of tables	25	25
Total number of elements	584	1617
Total number of attributes	395	787
Avg. number of elements	23.36	64.68
Avg. number of attributes	15.8	31.48
Max. number of elements	97	140
Max. number of attributes	23	64

## Data Availability

The data presented in this study are available on request from the corresponding author. The data are not publicly available due to copyright issues.
